# Connecting Age-Friendly University Principles to Undergraduate Education Builds Campus-Wide Buy-In

**DOI:** 10.1093/geroni/igaa057.1752

**Published:** 2020-12-16

**Authors:** Clare Luz

**Affiliations:** Michigan State University, East Lansing, Michigan, United States

## Abstract

Michigan State University became an Age-Friendly University (AFU) in December 2019. The path to campus-wide buy-in started years earlier by AgeAlive, a program established to serve as MSU’s central aging hub and to promote wellbeing throughout life. University approval and awareness of AgeAlive and what it means to be an AFU remains a key goal and crucial to sustainability. Central to strategic planning is ensuring that AgeAlive and MSU missions and values are aligned, visibility remains high, and outcomes are tangible and substantial. This presentation provides concrete examples of initiatives that meet these criteria and are building university support including a program that matches students to isolated seniors at risk for loneliness and multi-university research to help college students caring for older family members succeed as students and caregivers. Recommendations for developing and executing a strategic plan will be described and of interest to others on their own AFU journey.

